# Supplementary material for: Chimeric Peptides from Californiconus californicus and Heterodontus francisci with Antigen-Binding Capacity: A Conotoxin Scaffold to Create Non-Natural Antibodies (NoNaBodies)
Source: Toxins (Basel). 2023 Apr 4;15(4):269. doi: 10.3390/toxins15040269 (PMC10141372; doi:10.3390/toxins15040269)
Supplement: Supplementary file 1 [file toxins-15-00269-s001.zip › toxins-2270686-supplementary.pdf]

Supplementary Materials: Chimeric Peptides from *Californiconus californicus* and *Heterodontus francisci* with Antigen-Binding Capacity: A Conotoxin Scaffold to Create Non-Natural Antibodies (NoNaBodies)

Salvador Dueñas, Teresa Escalante, Jahaziel Gasperin-Bulbarela, Johanna Bernáldez-Sarabia, Karla Cervantes-Luévano, Samanta Jiménez, Noemí Sánchez-Campos, Olivia Cabanillas-Bernal, Blanca J. Valdovinos-Navarro, Angélica Álvarez-Lee, Marco A. De León-Nava and Alexei F. Licea-Navarro

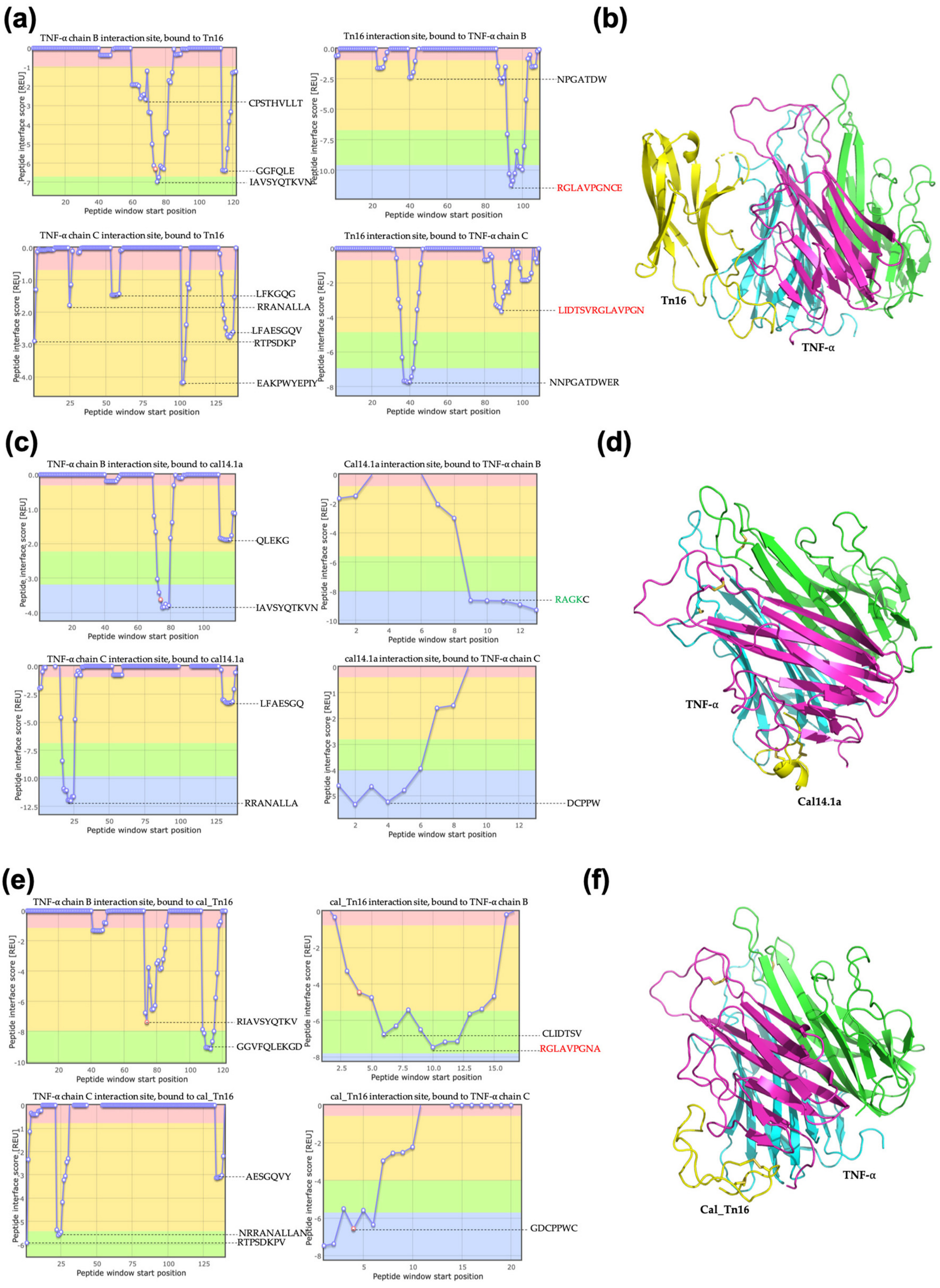

Figure S1. Protein-protein interaction analysis in ClusPro and Peptiderive web tool of different molecules against the TNF-α. a) Plot of the interaction regions of the positive control complex VNAR Tn16/ TNF-α. The sequence of complementarity-determining region 3 (CDR3) of variable domains of new antigen receptor (VNAR) Tn16 is shown in red. b) VNAR Tn16/ TNF-α complex, VNAR Tn16 (yellow cartoon), and TNF-α (chain A green cartoon, chain B cyan cartoon, and chain C magenta cartoon). c) A plot of the interaction regions of the negative control complex cal14.1a/ TNF-α. d) cal14.1a/TNF-α complex, cal14.1a (yellow cartoon), and TNF-α (chain A green cartoon, chain B cyan cartoon, and chain C magenta cartoon). e) Plot of interaction region of amino acids of a peptide cal_Tn16 against TNF-α. The CDR3 of the VNAR Tn16 in the scaffold appears in red. f) The cal_Tn16/TNF-α complex, NoNabody (yellow cartoon), and TNF-α (chain A green cartoon, chain B cyan cartoon, and chain C magenta cartoon).

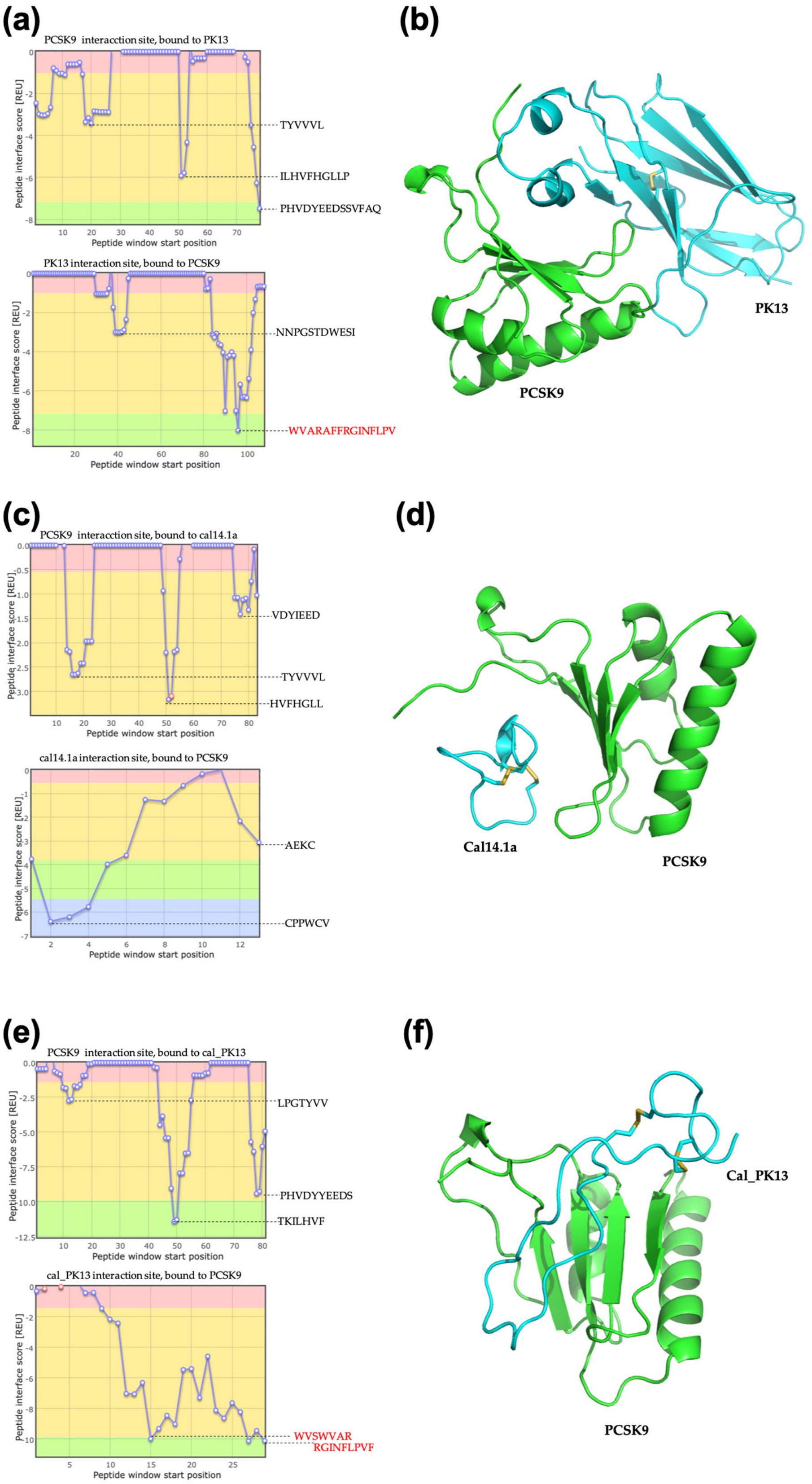

Figure S2. Protein-protein interaction analysis in ClusPro and Peptiderive web tool of VNAR PK13, cal14.1a, and cal_PK13 against PCSK9. a) A plot of the interaction regions of the positive control VNAR PK13 with PCSK9. The sequence of complementarity-determining region 3 (CDR3) of variable domains of new antigen receptor (VNAR) is shown in red. b) VNAR PK13/PCSK9 complex, PK13 (cyan cartoon), and PCSK9 (green cartoon). c) A plot of interaction regions of the negative control cal14.1a with PCSK9. d) Thee cal14.1a/PCSK9 complex, cal14.1a (cyan cartoon), and PCSK9 (green cartoon). e) A plot of the interaction regions of a NoNabody cal_PK13 with PCSK9. The CDR3 of the VNAR PK13 in the scaffold appears in red. f) cal_PK13/PCSK9 complex, cal_PK13 (cyan cartoon), and PCSK9 (green cartoon).

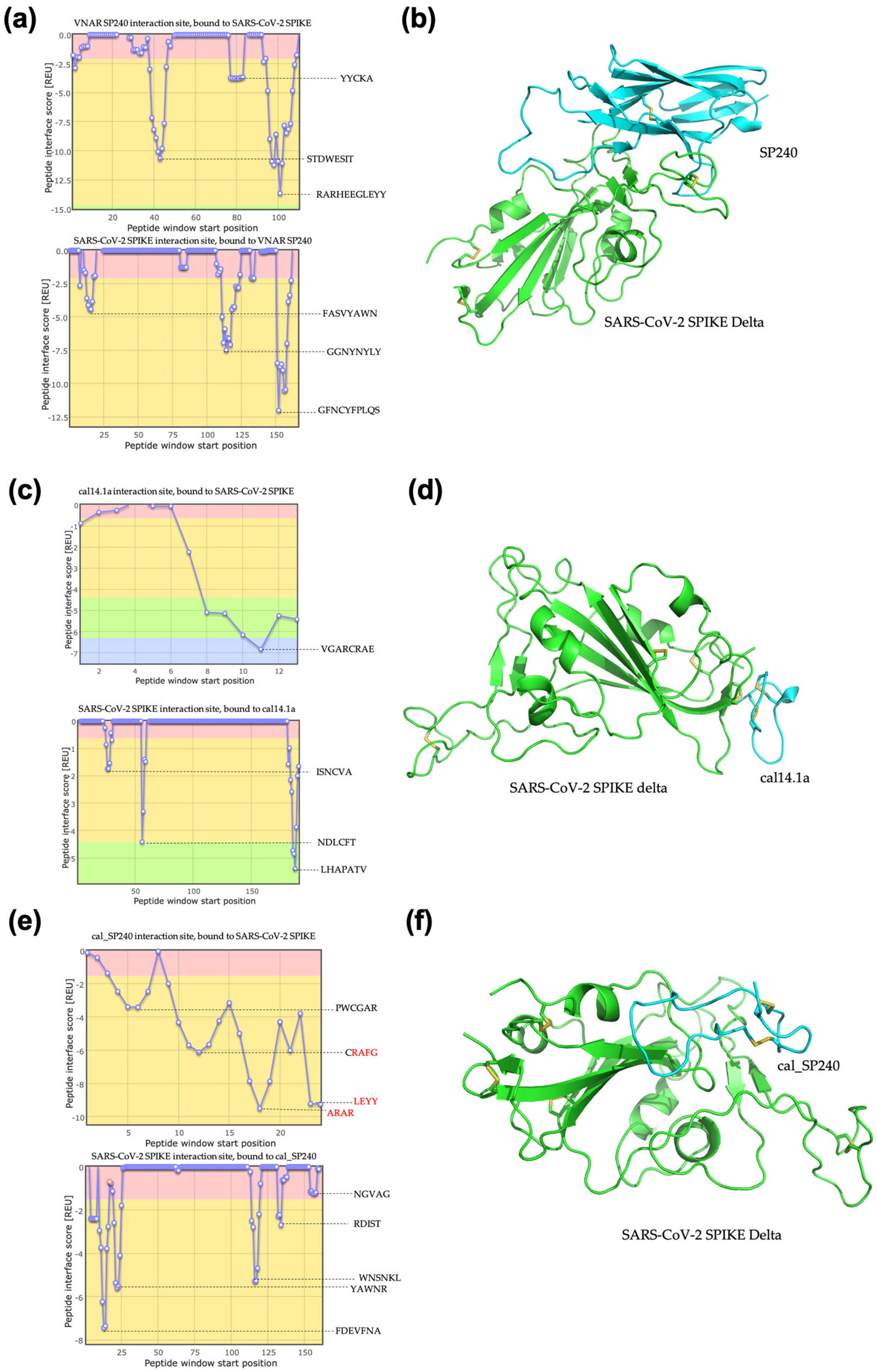

Figure S3. Protein-protein interaction analysis in ClusPro and Peptiderive web tool of different molecules against the SARS CoV-2 SPIKE. a) A plot of the interaction regions of the positive control complex VNAR SP240/SARS CoV-2 SPIKE. The sequence of the amino acids of CDR3 appears in red. b) VNAR SP240/ SARS CoV-2 SPIKE complex, VNAR (cyan cartoon), and SARS CoV-2 SPIKE (green cartoon). c) A plot of interaction regions of negative control. d) cal14.1a/SARS CoV-2 SPIKE complex, cal14.1a (cyan cartoon), and SARS CoV-2 SPIKE (green cartoon). e) Plot of interaction regions of cal_SP240 with SARS CoV-2 SPIKE. The CDR3 of the VNAR SP240 in the scaffold appears in red. f) cal_SP240/ SARS CoV-2 SPIKE complex, cal_SP240 (cyan cartoon), and SARS CoV-2 SPIKE (green cartoon).

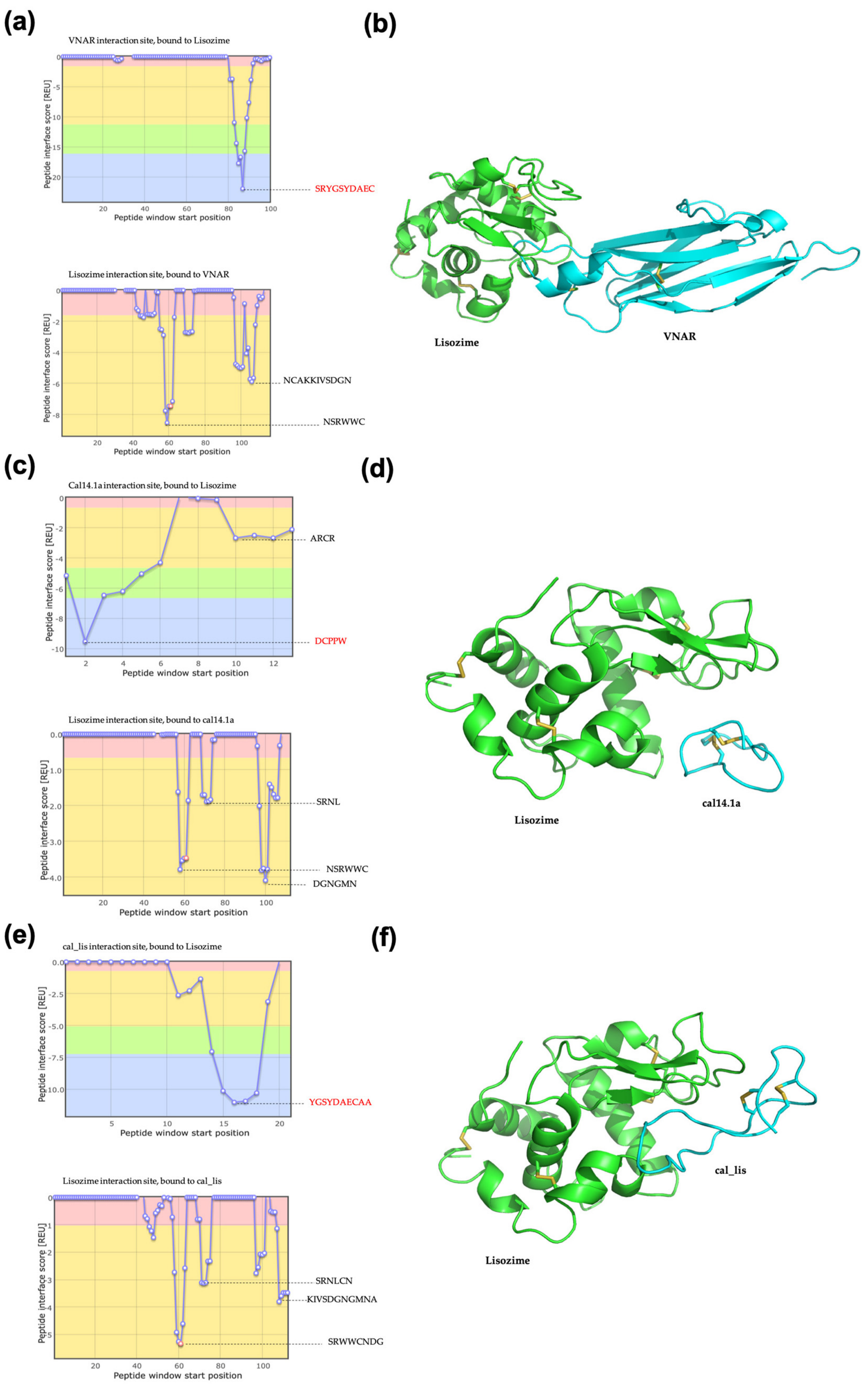

Figure S4. Protein-protein interaction analysis in ClusPro and Peptiderive web tool of different proteins against lysozyme. a) A plot of interaction regions of a variable domain of new antigen receptor (VNAR) from *G. cirratum* with lysozymes. The CDR3 of the VNAR is shown in red. b) VNAR (cyan cartoon) and Lysozyme (green cartoon). c) A plot of the interaction regions of negative control complex (cal14.1a/lysozyme). d) cal14.1a/ lysozyme complex, cal14.1a (cyan cartoon), and lysozyme (green cartoon). e) Plot of interaction regions of cal_lis with lysozyme. CDR3 of the VNAR in the scaffold appears in red. f) cal_lis/ lysozyme complex, cal_lis (cyan cartoon), and lysozyme (green cartoon).

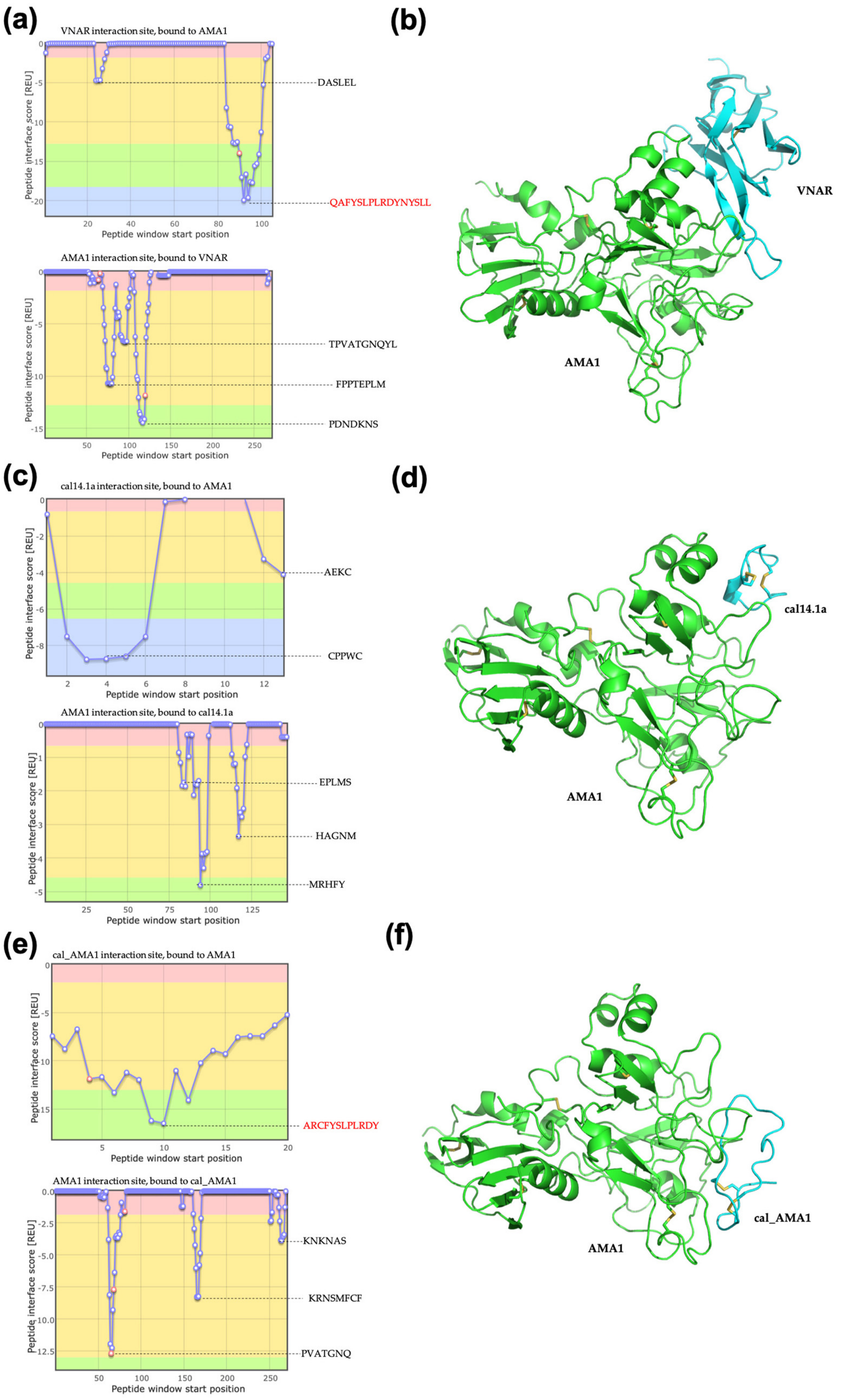

Figure S5. Protein-protein interaction analysis in ClusPro and Peptiderive web tool of VNAR (positive control), cal14.1a (negative control) and cal_AMA1 (NoNabody) against AMA1. a) A plot of interaction regions of a positive control complex. The complementarity-determining region 3 (CDR3) of variable domain of new antigen receptors (VNARs) appears in red. b) VNAR (cyan cartoon) and AMA1 (green cartoon). c) A plot of interaction regions of the negative control complex. d) cal14.1a (cyan cartoon) and AMA1 (green cartoon). e) Plot of interaction regions of the NoNabody with AMA1. The CDR3 of the VNAR in the scaffold is shown in red. f) cal_AMA1/AMA1complex, cal_AMA1 (cyan cartoon), and AMA1 (green cartoon).
